# Helminth Infections: Recognition and Modulation of the Immune Response by Innate Immune Cells

**DOI:** 10.3389/fimmu.2018.00664

**Published:** 2018-04-04

**Authors:** Claudia Cristina Motran, Leonardo Silvane, Laura Silvina Chiapello, Martin Gustavo Theumer, Laura Fernanda Ambrosio, Ximena Volpini, Daiana Pamela Celias, Laura Cervi

**Affiliations:** ^1^Departamento de Bioquímica Clínica, Facultad de Ciencias Químicas, Universidad Nacional de Córdoba, Córdoba, Argentina; ^2^Centro de Investigaciones en Bioquímica Clínica e Inmunología (CIBICI), Consejo Nacional de Investigaciones Científicas y Tecnológicas (CONICET), Córdoba, Argentina

**Keywords:** helminths, excretory-secretory products, phagocytes, M2 macrophages, dendritic cells, effector mechanisms, tissue repair

## Abstract

The survival of helminths in the host over long periods of time is the result of a process of adaptation or dynamic co-evolution between the host and the parasite. However, infection with helminth parasites causes damage to the host tissues producing the release of danger signals that induce the recruitment of various cells, including innate immune cells such as macrophages (Mo), dendritic cells (DCs), eosinophils, basophils, and mast cells. In this scenario, these cells are able to secrete soluble factors, which orchestrate immune effector mechanisms that depend on the different niches these parasites inhabit. Here, we focus on recent advances in the knowledge of excretory-secretory products (ESP), resulting from helminth recognition by DCs and Mo. Phagocytes and other cells types such as innate lymphocyte T cells 2 (ILC2), when activated by ESP, participate in an intricate cytokine network to generate innate and adaptive Th2 responses. In this review, we also discuss the mechanisms of innate immune cell-induced parasite killing and the tissue repair necessary to assure helminth survival over long periods of time.

## Introduction

Helminths, or worms, are invertebrate animals that comprise a broad spectrum of different pathogens able to affect human health. Among these parasites, there are two major phyla: the nematodes (or roundworms) and the platyhelminthes (also known as flatworms), with the latter in turn being subdivided into trematodes (flukes) and cestodes (tapeworms). The helminths infect a vast number of people all over the world, and it is estimated that soil-transmitted helminths cause infection in more than 1.5 billion people, or 24% of the entire human population. The main species that infect people are the nematodes *Ascaris lumbricoides, Trichuris trichiura, Necator americanus*, and *Ancylostoma duodenale*, with some of these producing chronic infections that can last up to 20 years (1).

The course of infection can vary greatly depending on the helminth. For example, certain filarial nematodes are transmitted by mosquitoes and can occupy and obstruct lymphatic vessels, which produces a chronic infection that causes lymphatic filariasis or elephantiasis (2), while other parasitic nematodes such as *T. trichiura* are strictly enteric and reside in the epithelium. In the case of schistosomiasis, an acute and chronic disease produced by different species of the trematodes worm *Schistosoma*, the pathology is caused by the inflammatory reaction of the host to the eggs deposited in the tissues. This triggers the development of granulomas constituted by an inflammatory infiltrate and fibrosis around the eggs. During the evolution of the granulomas, the excessive fibrosis can cause periportal hypertension and even occasionally strokes when its location is cerebral or spinal (3).

The survival of helminths in the host for long periods of time is the result of a process of adaptation or dynamic co-evolution between the host and the parasite. In the case of these pathogens, it is necessary for them to locate a niche suitable for maturation and propagation without killing or damaging the host. Otherwise, the host would be able to generate an effective immune response to expel the parasite or at least limit the negative fitness effects of a given parasite load by inducing tolerance without sacrificing its ability to respond effectively to other pathogens.

This review is focused on the mechanisms by which phagocytes, in particular macrophages (Mo) and dendritic cells (DCs), recognize helminth parasites, become activated and induce effector immune responses. We also discuss recent advances in the knowledge about how Mo, DCs, and other phagocytes, such as mast cells, Eos, and basophils, are involved in the shaping of the innate and adaptive immune responses. We analyze the protective immune mechanisms generated against helminths, with a particular emphasis being placed on the trematodes *Fasciola hepatica*.

## Helminth Recognition by Phagocytes: Modulation of the Immune Response by Secreted Products

Unlike other parasites, helminths are macropathogens, a condition that prevents them from being ingested by phagocytic cells. Thus, during infection by helminths, the products secreted by these parasites play a fundamental role as modulators of phagocyte activation by modifying the microenvironment in which these cells participate in the induction and instruction of the innate and adaptive immune responses.

The term “excretory/secretory antigens” (ES) refers to the parasite molecules that are released at the interface between the parasite and the cells of the immune system by various mechanisms, such as active secretion and diffusion from parasitic soma. These molecules are originated from adult worms intestinal content as well as female worms uterine content released during egg or larval deposition (4, 5). The development of systematic proteomic analysis has allowed many of the main ES helminth products to be characterized (6). These studies have revealed a common set of proteins that are secreted by helminths, including protease enzymes, glycolytic enzymes, protease enzyme inhibitors, antigens homologous to allergens, and lectins. However, as ES composition varies in different parasites and is affected by the stage of their life cycles, the reported different effects of ES on phagocytic cells may just be reflecting the complexity of their composition (5, 7).

Dendritic cells are mediators between innate and adaptive immunity, consequently, they play the principal role in the recognition, capture, processing, and presentation of helminth ES to T cells. A predominant Th2 response during helminth infections has been widely reported, although the precise mechanism initiating this response has not been fully elucidated (8). Nevertheless, it is clear that DCs are involved in the recognition of helminths or their products and the subsequent promotion of Th2 development. In fact, DCs are able to detect multiple ES by expressing the different innate immune receptors involved in the recognition of molecular patterns highly conserved in pathogens or PAMPs. Toll-like receptors (TLRs), C-type lectin receptors (CLRs), and nucleotide binding domain leucine-rich repeat (LRR)-containing (or NOD-like) receptors (NLRs) are the most studied of those involved in the interaction with helminth-derived molecules. DCs have been shown to recognize the *Schistosoma mansoni* lipid antigen containing phosphatidyl serine through TLR2, while lacto-N-fucopentaose III (LNFPIII) from *S. mansoni* and glycoprotein ES-62 of filaria are recognized through TLR4 (9, 10). In all cases, signaling through TLR2 or TLR4 reduces the ability of DCs to produce IL-12 and promotes a polarization toward a Th2-type response. This association between TLR signaling and polarization toward Th2-type response is surprising, as this signaling has been mostly associated with the development of a Th1-type response. However, it is important to highlight that the signaling cascade initiated in DCs downstream from TLRs, after their ligation by molecules derived from helminths, differs from that exerted by Th1 stimuli (such as LPS). Thus, the ligation of DC TLR4 by bacterial LPS strongly activates the mitogen-activated MAP kinases (MAPK) p38, JNK, and ERK, whereas the recognition of LNFPIII of *Schistosoma* by TLR4 present in DCs only induces phosphorylation of ERK (9). Similarly, the ES-62 protein of filaria and LNFPIII activate TLR4, but unlike LPS, they drive the response toward a Th2-type profile, suppressing the activation of p38 and JNK and inhibiting the production of IL-12 in an ERK-dependent manner. In the same way, the recognition of ES from eggs of *S. mansoni* through TLR2 is associated with the stabilization of the MAPK ERK, which promotes polarization to Th2 through the stabilization of the transcription factor c-Fos (which in turn suppresses the production of IL-12) (11). One possible explanation for these differences could be the association of the TLRs with different co-receptors, which may interfere with the downstream signaling cascade under TLRs. An example of this situation is the case of zymosan (an insoluble carbohydrate obtained from a yeast cell walls), which induces the production of the anti-inflammatory cytokine IL-10 by simultaneously signaling through the carbohydrate receptors dectin-1 and TLR2 (12). In fact, this mechanism of pattern-recognition receptors crosstalk could be occurring in the identification of carbohydrate helminths by the CLR. Consequently, it has recently been shown that the omega-1 molecule, a glycoprotein with ribonuclease activity secreted by *S. mansoni* eggs, signals *via* the mannose receptor (MR) through its carbohydrate domain. This recognition allows the internalization of omega-1 in DCs, with its RNAse activity being essential for inducing the Th2-type response (13). Other glycans derived from helminths, such as a Lewis^X^-containing glycan secreted by both the eggs and the schistosomula of *S. mansoni*, are recognized by DC-SIGN and MR receptors on DCs, while carbohydrates rich in N-acetyl galactosamine are recognized by the macrophage galactose-type lectin (4).

It is well known that for Th2 differentiation, the early production of IL-4 is essential. However, DC activation by helminth products fails to induce IL-4 secretion. Therefore, it can be assumed that the modulation of DCs to promote Th2 polarization is dependent not only on a direct effect of helminths or their products on these cells but also on cell interaction with tissue-derived factors, such as the “alarmins,” thymic stromal lymphopoietin (TSLP), matrix metalloproteinase 2 (MMP-2), IL-33, IL-25, and Eos-derived neurotoxin (EDN), among others, which also have an impact on DC activation [reviewed in Ref. (14)].

In contrast, to what was observed on DCs, the basophils and Eos have been reported to be involved in the development and amplification of the Th2 response, because of their ability to produce and secrete IL-4. Moreover, in addition to being key cells in the link between innate immunity and the development of the Th2 response, basophils, and Eos are phagocytes that are able to sense PAMPS as well as to process and present helminth antigens to naive T cells (15). Eos from healthy humans either treated with extracts from *Brugia malayi* or isolated from mice infected with this parasite have shown maturation signals with increasing MHC class II and some co-stimulatory molecule expression (16). In a similar way, *Strongyloides stercoralis* antigen*-*pulsed Eos induce Th2-type responses when injected into mice (17). Accordingly, Eos from MHC II-deficient mice fail to induce this type response, thereby highlighting the crucial role of these cells as antigen-presenting cells (APCs) in the induction of the Th2 adaptive immune response.

Basophils are granulocytes which represent about 1% of the circulating white blood cells. They are phagocytic cells able to induce inflammatory responses and share many characteristics with mast cells, such as the expression of the high-affinity Fc receptor for IgE (FcεRI) and the TLRs, TLR2 and TLR4. Basophils can also release mediators (including leukotrienes, prostaglandins, and histamine) that promote luminal fluid flow, nerve stimulation, and intestine contractility upon activation and IL-4 secretion (18). Although basophils are an important source of IL-4 and IL-13, they have been shown to be dispensable in the generation of the Th2 response in some helminth infections [e.g., during *Nippostrongylus brasiliensis* infection in basophil-deficient mice (Mcpt8-cre)]. Th2 polarization occurs even in the absence of these cells (19). By contrast, injection of mice with IPSE/alpha-1 protein derived from *S. mansoni* eggs induces IL-4 production by basophils, which contributes to initiating a Th2-type response, emphasizing the importance of basophils IL-4 in the linking of innate immunity and Th2 development (20). Recent studies have proposed that basophils can act as APCs, and, in this regard, the ability of basophils as APCs to promote the Th2-type response against helminth parasites might be dependent on MHC II expression. However, two controversial points arise from different studies proposing basophils as APCs: first, the low expression levels of the H-2M invariant chain, crucial in the regulation of the MHC-II peptide loading and, second, the low levels of MHC II expression in these cells compared to professional APCs. These issues have been clarified recently, since basophils can obtain peptide–MHC-II complexes from DCs by trogocytosis, allowing these cells to function as APCs, promoting a Th2-type response (21).

Mast cells originated from bone marrow, enter the peripheral blood, and complete their differentiation in tissues such as the skin or gut. A relevant study from Hepworth et al. demonstrated the involvement of mast cells in the development of the innate and adaptive Th2 responses during helminth infection (22). This study showed that in the absence of mast cells, mice infected with *Heligmosomoides polygyrus bakeri* or *Trichuris muris* revealed a deficiency in the production of the alarmins IL-25, IL-33, and TSLP derived from tissues. This led to an impaired innate and adaptive Th2-type responses; thus, showing the relevance of mast cell-induced responses during the early stages of gastrointestinal helminth infection.

Interestingly, Eos, basophils, and mast cells can also modulate and instruct the adaptive immune response after their stimulation through the release of the granules’ content, as will be described below.

## Modulation of Phagocyte Activation by *F. hepatica*

*Fasciola hepatica* is a causative agent of fasciolosis, which is a neglected disease that affects a vast number of cattle and sheep throughout the world and now is becoming an emerging disease in humans (23). During its migration through the host tissues, the parasite excretes and/or secretes many products capable of modulating the immune response. Soon after infection, the larval stage of *F. hepatica* crosses the intestinal wall and reaches the peritoneum. At the same time, the recruitment of alternatively activated Mo occurs, with upregulation in the expression of Fizz, Ym1, and arginase-1.

**Figure 1:**
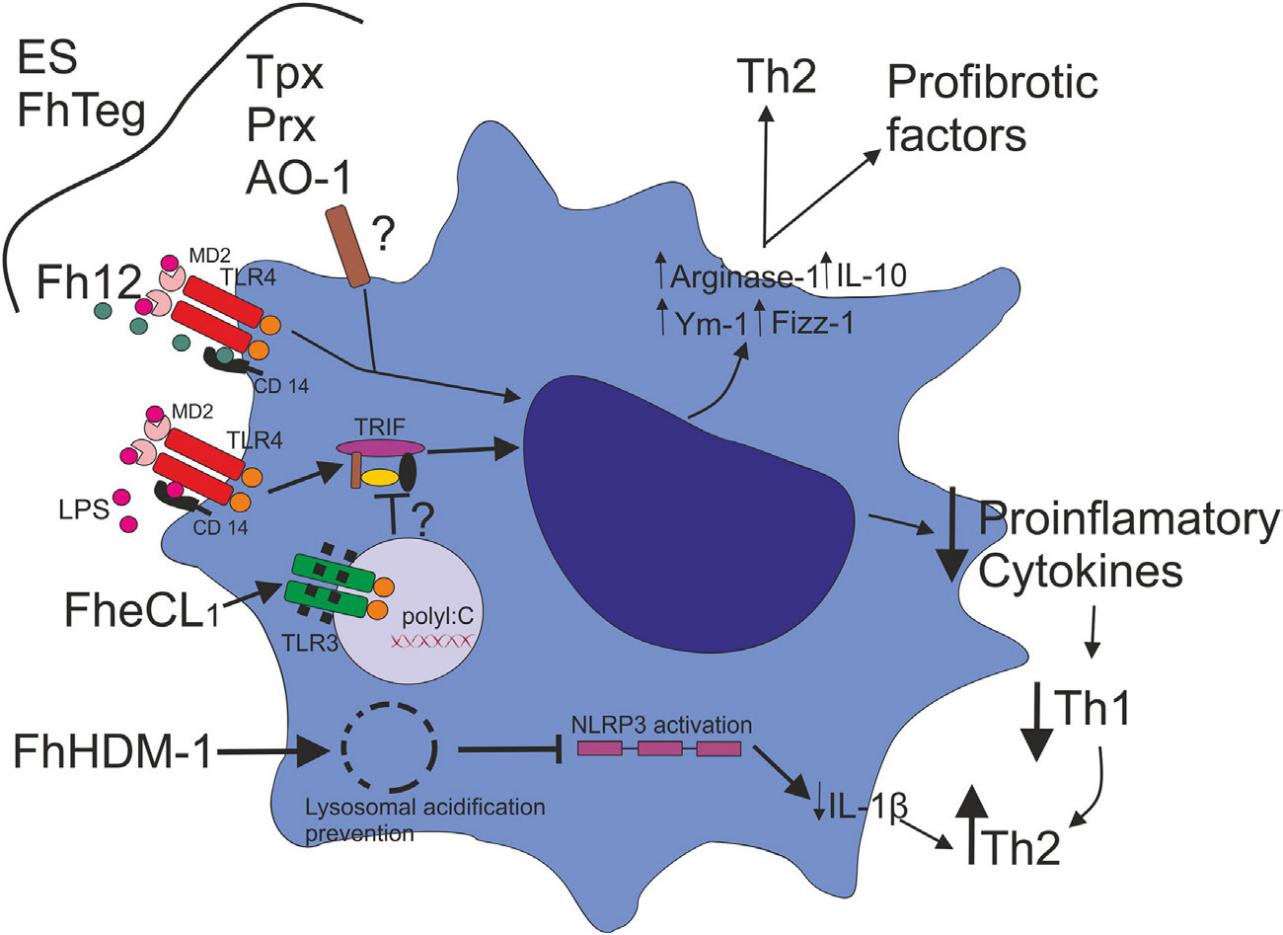
*Fasciola hepatica* modulates macrophage activation*. F. hepatica* ES, *F. hepatica* tegumental antigens (FhTeg), and ES-derived enzymes, such as thioredoxin peroxidase (Tpx), 2-Cys peroxiredoxin (Prx), and heme-oxygenase-1 (AO-1) drive Mo phenotype toward an alternative activation profile. Fatty acid binding protein (Fh12) binds the co-receptor CD14 and inhibits LPS-induced TLR4 activation, suppressing the expression of IL-12, TNF, IL-6, and IL-1β. *F. hepatica* Cathepsin L1 (FheCL1) induces TLR3 degradation, which impairs LPS-induced TLR4–TRIF-dependent pathway with the consequent inhibition of inflammatory cytokine production. The decrease of an inflammatory cytokine milieu might promote the inhibition of a Th1 type response favoring a Th2 profile. *F. hepatica* defense molecule 1 (FhHDM-1) has been shown to have the ability to destabilize lysosomal acidification, which impairs Mo NLRP3 activation and consequently inflammasome function, resulting in the downregulation of IL-1 β production.

Many investigations have shown the ability of *F. hepatica* ES (24), *F. hepatica* tegumental antigens (FhTeg) (25), and ES-derived enzymes [such as thioredoxin peroxidase (26), 2-Cys peroxiredoxin (27), fatty acid binding protein (28) and more recently heme-oxygenase-1 (29)] to modulate Mo phenotype toward an alternative activation. This profile promotes three fundamental effects: the secretion of anti-inflammatory factors, the driving toward a Th2-profile and an increase in the pro-fibrotic factors involved in wound healing (Figure 1) (30). These are essential steps to compensate for the damage caused by the migration of the parasite in the host tissues, thereby allowing its establishment in the liver during the chronic phase of the disease.

Interestingly, a secreted peptide from *F. hepatica* called helminth defense molecule 1 (FhHDM-1) has been shown to have the ability to destabilize lysosomal acidification, which impairs Mo NLRP3 activation and consequently inflammasome function, resulting in the downregulation of IL-1 β production. These authors propose that in the absence of a Th1-type inflammatory milieu, the Th2-type immune response and parasite survival is favored (Figure 1) (31).

In addition, a mechanism of inhibition of TLR-dependent Mo activation was described by Donnelly et al. In this study, the cysteine protease activity of *F. hepatica* Cathepsin L1 (FheCL1), has been involved in the degradation of TLR3 within the endosome upon TLR3 and TLR4 stimulation. The ability of FheCL1 to inhibit TRIF-dependent signaling is a strategy used by *F. hepatica* to control innate immune responses suppressing consequently Th1 development (Figure 1) (32).

Similarly, the exposure of DCs to *F. hepatica* products inhibits the ability of these cells to mature in response to inflammatory stimuli. Related to this, we have shown that *F. hepatica* ES inhibits TLR-activated DCs maturation and their ability to induce allogeneic responses (33). FhTeg, isolated from the coat of the parasite, has also been reported to downregulate inflammatory cytokine production and the expression of co-stimulatory molecules in response to TLR and non-TLR stimuli (34).

The relevance of the modulatory effect of *F. hepatica* antigens on DCs was demonstrated by the ability of these cells to inhibit inflammatory responses, such as those observed in the collagen-induced arthritis model (CIA). We evaluated the capacity of DCs treated with a total extract (TE) of *F. hepatica* together with CpG to modulate the inflammatory responses in CIA. The immunization of mice with collagen II-pulsed TE/CpG-conditioned DCs to diminish the severity and incidence of CIA symptoms induced anti-inflammatory cytokine production and promoted regulatory T cell (Treg) development (35). In addition, we have identified a protein Kunitz-type molecule that is present in TE as being able to inhibit TLR-induced DCs activation (36). Overall, these findings suggest that the modulation of DC activation might aid the chronic establishment of the parasite in the host.

In recent years, there has been an important advance in the knowledge about the receptors involved in the recognition of *F. hepatica* products. The role of CLRs, such as MR and dectin-1 in the interaction with *F. hepatica* ES has been reported. A partial inhibition of immunomodulatory factors such as arginase-1 expression, with TGF-β and IL-10 production has been observed when these receptors were blocked (24). In line with these findings, it was demonstrated that the recognition of FhTegs by MR-DCs is essential for the induction of Foxp3+ Treg cells and the CD4+ T cells anergy. This study showed that in the absence of MR, the ability of DCs to induce T cell anergy markers is downregulated (37). Finally, the participation of the CLR DC-SIGN, which recognizes *F. hepatica* glycans, has recently been demonstrated. In fact, DC-SIGN triggering is a critical event that induces some DC regulatory functions, such as the ability of these cells to induce anergic/Treg cells (38). Overall, all these results highlight the importance of improved knowledge about innate immunity receptors such as C-type lectins in the recognition and decoding of helminth molecular patterns, which are relevant to the induction of immunoregulatory responses.

## Killing of Helminths Mediated by Phagocytes and Antibodies

**Figure 2:**
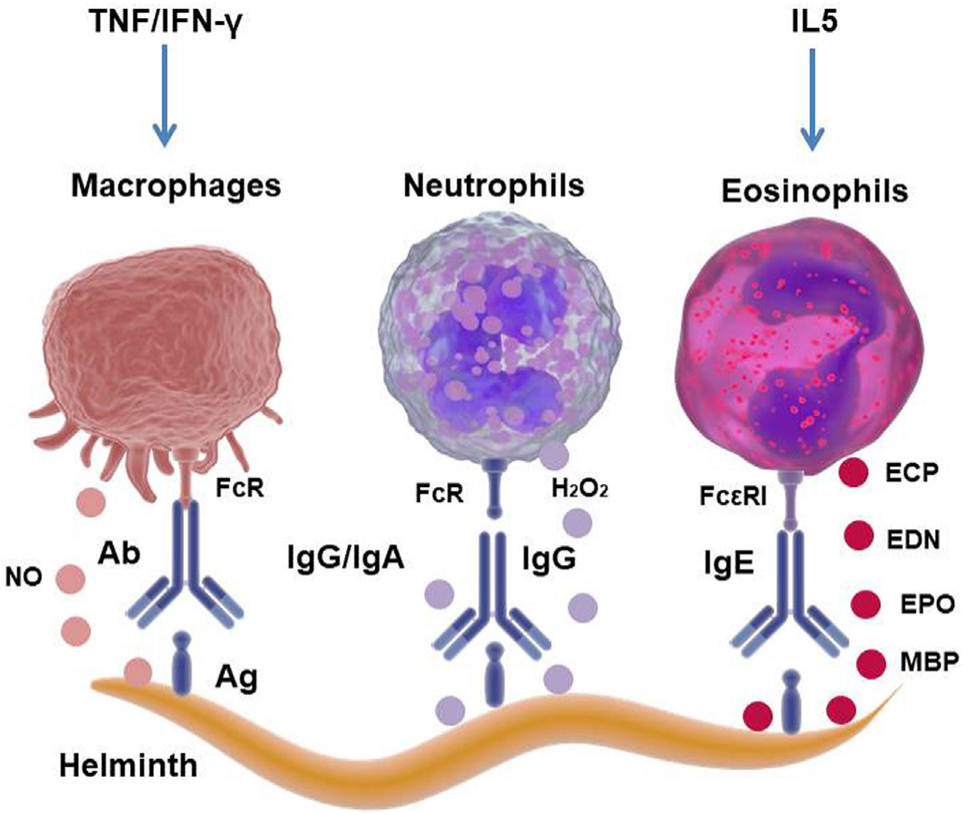
Mechanism of cellular cytotoxicity mediated by antibodies against helminth parasites. The interaction of the antibodies that cover the parasite with the Fc receptors present in eosinophils (Eos), neutrophils, or macrophages (Mo) induces degranulation and the release of lysosomal/granular content, hydrogen peroxide (H_2_O_2_) or nitric oxide (NO), causing lysis of the helminth. Eos granules contain parasite toxic proteins, such as major basic protein (MBP), eosinophil peroxidase (EPO), eosinophil cationic protein (ECP), and eosinophil-derived neurotoxin (EDN).

Among the cells that belong to the innate immune system, in the first line of defense against microorganisms, the phagocytic cells mediate the internalization and destruction of some pathogens, especially those that are opsonized by antibodies. As mentioned above, as helminth parasites are macropathogens, they cannot be ingested by phagocytic cells. Therefore, the immune system uses other mechanisms in order to eliminate them, such as antibody-dependent cellular cytotoxicity (ADCC), which is also referred to as antibody-dependent cell-mediated cytotoxicity. The Fc-receptor-bearing effector cells can recognize and kill antibody-coated parasite worms by discharging their lysosomal or granular content (15) (Figure 2).

As stated above, helminth infections strongly induce Th2-skewed responses associated with cytokines (e.g., IL-4, IL-5, and IL-13), mastocytosis, eosinophilia, and antibody class-switching producing IgE [reviewed in Ref. (39)]. This antibody isotype is greatly elevated in helminth infection and mainly involved in ADCC. As a result, it is considered almost certain that the contribution of Eos to the host defense against helminth parasites is crucial (40). In agreement with this, *in vitro* studies have demonstrated that Eos use ADCC mechanisms to directly eliminate a variety of helminthic species through the action of Eos granule proteins. Furthermore, *in vivo* studies have shown that Eos can directly kill helminthic larvae (41, 42), exept for the adult forms of the parasites, which are more relevant to the infection (43).

Antibody-dependent cellular cytotoxicity by Eos degranulation occurs as a consequence of the interaction of Eos FcεRI with the Fc portion of specific-IgE bound to the parasite. The Eos granules mainly contain cytotoxic proteins such as the major basic proteins (MBP-1 and MBP-2), eosinophil peroxidase, eosinophil cationic protein (ECP) and EDN, which cause cytolysis of the parasite when released into the extracellular space (Figure 2) (15, 44). Mouse models of Eos depletion have demonstrated the protective role of these cells in secondary infections with helminths, with the depletion of Eos inducing an increase in the parasite burden of *N. brasiliensis* and *Trichinella spiralis* in murine models (45). Moreover, it has recently been reported that Eos are an important source of growth factors for plasma cell survival, suggesting an important role in the effector function to sustain antibody production (40). Eos produce cytokines and chemokines, such as IL-4, IL-5, IL-13, TGF-β, IL-10, IL-8, IL-12, IL-18, TNF, CCL11, and CCL5, with these cytokines being stored pre-formed in the granules to be rapidly released after Eos degranulation (46). Thus, these molecules can rapidly modulate innate and adaptive immune responses, and emerging data have demonstrated that during *T. spiralis* infection, Eos favors parasitic infection by inhibiting the Th1-type response (47). Therefore, it is questionable whether Eos contribute to anti-helminthic immunity. Furthermore, it is possible to conclude that eosinophilia is a sign of helminthic infection and that Eos are critically involved in immunoregulatory and/or host protective effects during parasitic worm infections.

Macrophages from rats have been involved in the *in vitro* ADCC of newly encysted juvenile (NEJ) worms of the trematodes *F. hepatica* through nitric oxide-dependent killing (Figure 2). Similarly, Mo and Eos from sheep have been shown to be able to eliminate juvenile larvae from *F. gigantica* through the release of superoxide radicals (48). However, this latter mechanism was shown to be ineffective when ovine peritoneal cells were cultured with *F. hepatica* NEJ, suggesting the ability of this parasite to resist elimination by reactive oxygen species, possibly through the release of antioxidant enzymes (48).

The predominant cell in the early response to schistosomula is the neutrophil, and the earliest histologic signs of parasite damage involve larval–neutrophil interactions (49). Besides, the killing of schistosomula by neutrophils (50, 51) or Mo (52, 53) has been reported. Also, ADCC by lung cells from rats infected with *T. spiralis* was demonstrated to be effective against newborn larva (NBL) with the FcεRI receptor present in Mo and neutrophils with Eos playing a critical role in cytotoxic attack (54). In addition, Bass and Szejda reported that neutrophils are at least as effective as Eos in the killing of NBL of *T. spiralis*, which appeared to be mediated by the generation of hydrogen peroxide (55).

Despite considerable pieces of evidence from *in vitro* assays showing the killing of helminth parasites by ADCC (15), there are few reports about whether this mechanism occurs *in vivo*. However, it has been demonstrated in helminth infections that the absence of antibodies or effector cells, crucial in ADCC, has a significant impact on the resistance to infection. In this regard, depletion of Eos, neutrophils, or Mo in studies of mice infected by helminths has revealed the importance of these cells in controlling infection levels (44, 56). In a similar way, antibody production was shown to correlate with protection in mice helminth infections (57, 58). In agreement with this, mice lacking antibodies (J_H_^−/−^) or activating Fc receptors (FcRγ^−/−^) have shown increased numbers of larvae when infected with *Heligmosomoides polygyrus bakeri*, highlighting the relevant role of antibodies in the protective mechanism against helminth parasites (59).

Some mechanisms identified in helminth infections to prevent the immune system attack have been reported. One example that shows an inefficient ADCC is the ability of *F. hepatica*, on the one hand, to cleave IgG and IgE through the release of cathepsin L-protease and, on the other hand, to induce apoptosis of Eos and Mo (60–62). Furthermore, the release of antioxidant enzymes such as superoxide dismutase or glutathione transferase could inhibit the superoxide anion, which is potentially toxic for the parasite larvae (63). It has also been recently demonstrated that a TGF-β-like molecule from *F*. *hepatica* called FhTLM, which is expressed by the NEJ stage of the parasite, inhibits the ability of Mo-dependent ADCC by using the TGF-β receptor (64). In this way, the parasite is able to inhibit each of the different components of the ADCC mechanism (such as effector cells, antibodies, and toxic molecules), resulting in its escape from the immune system and allowing its survival in the host.

Early studies by James et al. and Moser and Sher, have shown the ability of different granulocytes such as Mo, Eos, and neutrophils to kill the schistosomula. These granulocytes play a critical role in the antigen-antibody specific interaction on the surface of the larvae with the resulting death of these parasites (65, 66). In line with these findings, CBA mice immunized with an irradiated cercariae showed subdermal inflammatory foci around the dead *Schistosoma* larvae, with eosinophils and mononuclear cells the main infiltrating cells (67).

Mast cells are inflammatory cells that respond to different signals from innate and adaptive immunity by the release of inflammatory mediators. Thus, the phenotype and function of mast cells is modified by Th2-type cytokines, such as IL-4 and IL-5. Activation of mast cells is associated with the aggregation of FcεR1 by the antigens recognized by bound IgE. The density of FcεR1 on these cells is upregulated by higher levels of free IgE or the presence of IL-4 (49). Moreover, mast cells are activated by TLR ligands through damage-associated molecular patterns and the binding of IgG to FcγR1 and C3a and C5a through CD88. An example of the effector function of mast cells against helminths is their participation in IgE-mediated immediate hypersensitivity reactions, for example, against enteropathogens (49), thereby contributing to diarrhea production and at the same time assisting in parasite expulsion. The immediate hypersensitivity is a consequence of the presence of high levels of non-specific IgE generated by the Th2-type cytokine milieu as a result of worm infection. This cytokine binds FcεRI on mast cells, inducing degranulation (anaphylactic degranulation) and the release of inflammatory factors when they are cross-linked by cross-reactive antigens on the surface of the larva or adult worms (49, 68). Intriguingly, ES-62, a molecule secreted by filarial helminths, is able to inhibit the FcεRI-induced release of allergy mediators by selectively blocking key signal transduction events in mast cells, thus protecting the mice from cell-dependent hypersensitivity (69).

## Mo in Helminth Infections: Recruitment, Activation, and Control of Tissue Damage

A subject of recent controversy is related to the origin of the Mo that expand during helminth infections. In the case of viral, bacterial, or fungal infections and even protozoa, the Mo of tissues have been reported to be derived from monocytes. However, it is not that clear this is the case during helminth infections. As regards the origin of the Mo, there is a report showing that in infections by nematodes, such as *Litomosoides sigmodontis*, an inflammatory infiltrate rich in Mo in the pleural cavity appears because of the proliferation of these cells due to the effect of IL-4, rather than by a recruitment of monocytes (70). By contrast, during *S. mansoni* infection in mice, the majority of Mo in the liver was demonstrated to be monocyte-derived, with CCL2 being involved in the recruitment process (71). A possible explanation for this difference could be the fact that in the schistosomiasis model, the classical inflammation due to TLR activation can occur due to the leakage of bacterial ligands from the intestinal lumen to the circulation, with the resulting passage of the eggs from the vasculature to the lumen. In contrast, in infection with *L. sigmodontis*, which resides in sterile tissues such as lung, the recruitment of Mo to the pleural cavity is mainly a consequence of local Mo proliferation (70).

Tissue damage caused by the migration of helminth parasites through different organs of the host triggers a “type-2” inflammatory response characterized by the recruitment of cells, including basophils, Eos, and Th2-type CD4 lymphocytes. The secretion of cytokines IL-4 together with IL-13 from these cells promotes the accumulation and activation of Mo toward an M2 (also known as alternatively activated phenotype). Numerous examples of murine models of helminth infection (*S. mansoni, H. polygyrus, N. brasiliensis, Tenia crassiceps, T. spiralis, L. sigmodontis, F. hepatica, Ascaris suum*, and the filarial parasite *B. malayi*) show the recruitment of M2 Mo after a few days of infection (30).

Interestingly, it has recently been demonstrated that alternative activation of Mo could be induced in an antibody-dependent and an IL-4Rα-independent way. Thus, during infection with the murine parasite *H. polygyrus bakeri*, helminth antigens can develop a new type of Mo called helminth-antibody activated macrophages, which are involved in the resistance mechanism against the helminth and in the avoidance of tissue damage (59).

Different markers such as IL-4Rα, MR (CD206), and arginase-1 activity together with its metabolic products (such as urea and proline) are characteristic of M2 Mo (30). In addition, soon after infection with helminth parasites, the M2 Mo produce high levels of the proteins Ym1 and Fizz, which are both secreted after the activation of these cells by Th2 cytokines (72). Ym1, a lectin with an affinity for chitin, is a member of mammalian proteins that share homology with chitinase family proteins and has been described as an Eos chemotactic factor (73, 74). A role in cell-to-cell and cell-to-extracellular matrix (ECM) interactions has been attributed to Ym1 due to its ability to bind heparin (75), and deposit ECM involved in the wound healing process (73).

Another abundant protein secreted after a nematode infection, Fizz1 (also known as resistin-like molecule α) has also been implicated in the process of deposition of ECM (76), which has angiogenic properties and the capacity to stimulate actin and collagen synthesis. M2 Mo also promote increased levels of vascular endothelial growth factor, insulin-like growth factor 1 (IGF-1), and MMPs, and trigger receptor expression on the myeloid cells 2 (TREM2), TGF-β and growth factors such as platelet-derived growth factor (PDGF). All these factors have been shown to be involved in different stages of the wound healing responses. Also increased levels of these mediators have been reported in helminth infections, suggesting that they might contribute to the control of the tissue damage induced by these parasites (77–79).

The early stage of tissue repairing (up to hours or few days after tissue damage occurs) involves events including bleeding and inflammation with the recruitment of mast cells, neutrophils, platelets, as well as monocytes, which play a role in the control of bleeding. Different events of tissue damage and hemorrhages take place during the life cycle of *S. mansoni* as follows:

1. The cercariae penetrate the skin of the host by means of the secreted proteases.
2. Immature schistosomula can cross capillaries to enter the arterial flow.
3. Adult parasites mate in the mesenteric veins and the female lays eggs.
4. The eggs from the veins enter the gut wall and some reach lumen.
5. Some eggs are dragged by the blood flow into the liver where they are trapped in the small sinusoidal vessels, inducing a strong granulomatous response.

Thus, all stages of the parasite produce relevant tissue damage and hemorrhages in the host (78). Another example of tissue damage occurs during the life cycle of *F. hepatica*, where NEJ cross the intestinal wall, fall into the peritoneum and then go through the liver parenchyma leaving tunnels that cause necrosis (80). Among the nematodes, *A. lumbricoides* and *N. brasiliensis* cause damage to the lung tissue during their migration through the host (15).

In the healing process after tissue damage, the proliferation of fibroblasts and remodeling takes place. In particular, neutrophils and Mo are important cells because of their phagocytic capacity to help in debris removal. In addition, Mo and fibroblast induce fibroblast proliferation and promote fibrogenesis and collagen production through the release of different molecules, such as TGF-β and PDGF (81, 82). Interestingly, an opposing role can be attributed to Mo during an inflammatory process after tissue damage. On the one hand, these cells are able to activate a pro-fibrotic process by promoting the recruitment of inflammatory cells, which stimulate the activity of fibroblasts and the deposition of ECM (83). On the other hand, Mo are able to terminate the inflammation by the elimination of the debris coming from the rupture of tissue including dead inflammatory cells (82).

Tissue damage can cause upregulation of the alarmin IL-33. This is a strong positive stimulus for innate lymphocyte T cell 2 (ILC2) accumulation and cytokine production, which can support tissue-protective M2 Mo differentiation. Recent data suggest the possibility that ILC2, M2 Mo, amphiregulin (AREG), and IGF-1 may collaborate in promoting wound healing at the mucosal barrier surfaces.

Interestingly, Mo are also involved in the immunopathology of schistosomiasis, since in both mice and humans infected with *S. mansoni* many of these cells are recruited to the granulome in response to trapped eggs in the liver, intestinal or bladder tissues. In a mouse model of *S. mansoni*, it was demonstrated that Mo are the predominant cell population in gut granulomas, in contrast to what was observed in the liver, where Eos are the majority of the cells recruited (15).

In addition to promoting tissue repair, M2 Mo also play a role in the inhibition of the pro-inflammatory responses mediated by M1 Mo and Th1 and Th17 responses, which in turn can exacerbate tissue damage if not controlled. A demonstrative study from Ref. (84) shows that IL-4Rα signaling in Mo is essential for mice survival during acute schistosomiasis. Thus, the absence of IL4R-α in Mo was responsible for the death of mice due to increased levels of Th1 cytokines, NOS-2 activity and immunopathology in the liver and gut as well as increased egg expulsion. These results highlight the key role played by M2 Mo in protection against tissue damage. In the same line, results from a human study show the importance of the Th2 response to down modulate the inflammatory response in acute schistosomiasis (85). In addition, an Arg-1 Mo-dependent suppressive mechanism to control excessive fibrosis (characteristic of the severe human disease) has been recently reported. However, this is a surprising finding, considering that Arg-1 is involved in the synthesis of proline (amino acid precursor of collagen), and therefore in the promotion of fibrosis and tissue repair. At the same time, Arg-1-derived M2 Mo help in the resolution of Th2-type-dependent inflammation and fibrosis by acting as suppressor cells (86).

## Role of Phagocytes during Orchestration of the Intestinal Immune Response in Helminth Infections

Intestinal helminth parasites interact with the host intestinal barrier producing damage related to different causes, such as the attachment of the parasite to epithelial cells, migration through the host tissue, worm feeding events, or secondary opportunistic bacterial infections (87). Damage to host tissues induces the release of alarmins including the cytokines IL-33, IL-25, and TSLP by intestinal epithelial cells (IECs), among others. Furthermore, helminth infection promotes the early recruitment of the phagocytes Eos, mast cells, and basophils at the site of infections, which provide the rapid secretion of the Th2-type cytokines IL-4, IL-13, and TSLP (88). In addition to IECs, Mo, Eos, neutrophils, and mast cells can also secrete endogenous inflammatory mediators or alarmins, which can function as chemotactic factors or induce DC maturation (88, 89). These alarmins include EDN, cathelicidins, defensins, and high-mobility group box protein 1 with a suggested role of EDN in the initiation and maintenance of the Th2-type response (90). However, the mice models using anti-IL-5 or anti-CCR3 antibodies for Eos depletion have shown the dispensable role of these cells in the promotion of the Th2 response against helminth parasites (91).

A dominant Th2-type cytokine response in infected hosts influences the development of intestinal and systemic immune and immunopathological changes. These include strong peripheral and intestinal IgE-type responses, eosinophilia, goblet cell (GC) hyperplasia, villous atrophy and crypt hyperplasia, increased muscle contractility, and mastocyte hyperplasia in the intestinal mucosa. In addition, profound changes in the function of the IECs have been observed, including alterations in permeability, proliferation, and differentiation (92). All these mechanisms together contribute to the expulsion of the parasite from the intestinal lumen.

The hypercontractility of the intestinal smooth muscle in an IL-4/IL-13-dependent manner is a characteristic of nematode infections. Furthermore, the involvement of M2 Mo in the regulation of intestinal contractility has been demonstrated. The expulsion of worms from the lumen appears to be mediated by the expression of Arg-1 in M2 Mo, as a regulator of this function (93). By contrast, innate lymphoid cells (ILCs) perform crucial functions in different tissues, particularly at the mucosa surface of the intestine and lung, where they are important regulators of the innate immune response. Among these, the ILC2s are localized mainly in mucosa-associated tissues with IL-25 together with IL-33. These innate immune responses contribute to the elimination of parasites such as *N. brasilienses* (a gastrointestinal nematode that infects mice and has a life cycle similar to that of hookworms in humans) (94). In line with this, it has also been reported that IL-33 has a crucial role in resistance against other nematodes including *S. venezuelensis* in mice (95) and *T. muris* (96). After being activated by IL-33, the ILC2s increase their IL-13 production, which in turn mediates two mechanisms that contribute to the rodent hookworm *N. brasiliensis* being expelled from the intestinal lumen:

(1) The induction of the anti-nematode protein resistin-like molecule beta or Fizz2 (RELMβ) expression, a protein released by GCs which are able to inhibit worm nutrition (97).
(2) Eos recruitment, a key cell for eliminating intestinal nematodes from infected hosts.

**Figure 3:**
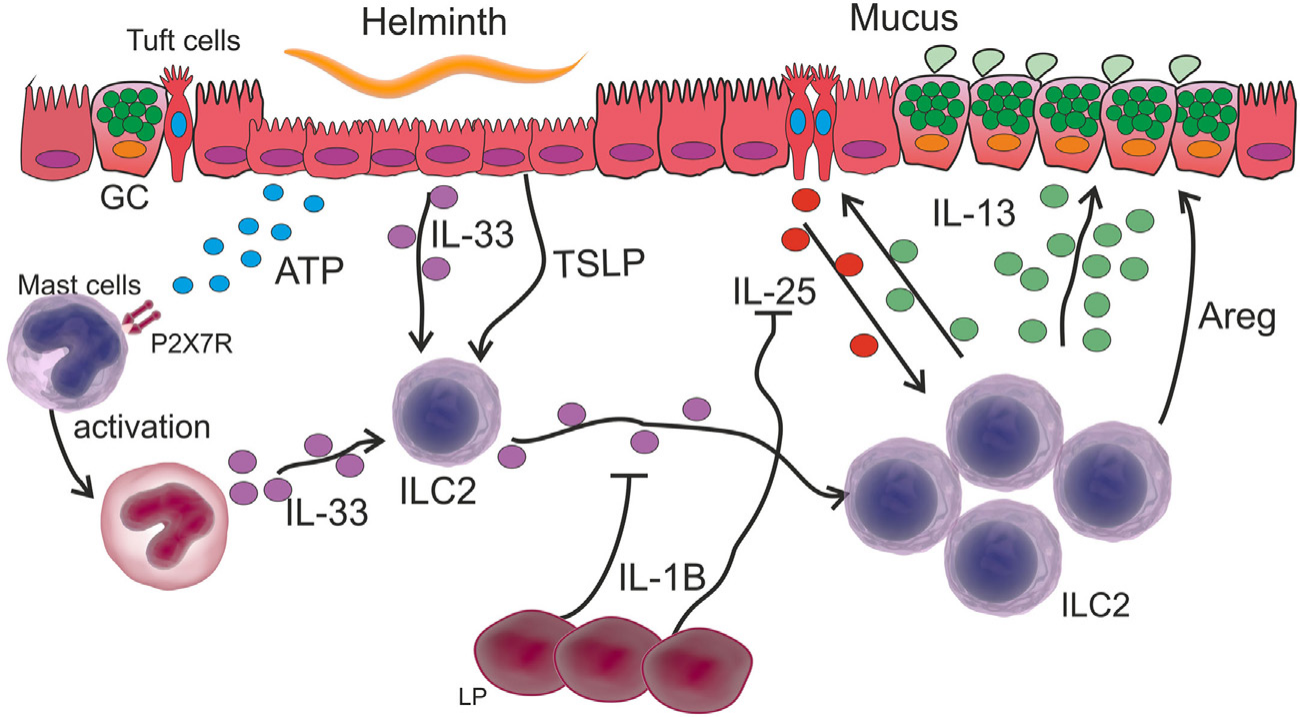
Innate immune response in intestinal helminth infection. Tissue damage caused by intestinal helminths induces an ATP increase that is recognized by P2X7R on the surface of mast cells, which in turn are activated secreting IL-33. This cytokine together with other alarmins such as stromal lymphopoietin (TSLP) activate ILC2 and induce their proliferation. Activated ILC2 cells secrete IL-13 thereby increasing mucus secretion in goblet cells (GC). The activation of ILC2s induces the hyperplasia of tuft cells and the release of IL-25, which together with IL-33 promotes the secretion of IL-13 by ILC2 cells. IL-1β produced by lamina propria (LP) cells inhibits IL-25 and IL-33, thereby controlling the expansion of the Th2-type response.

Interestingly, after *N. brasiliensis* infection, the activation of ILC2s induces the hyperplasia of tuft cells (a secretory intestinal cell type), which in turn are capable of secreting IL-25, and together with IL-33 promotes the secretion of IL-13 by ILC2 cells (Figure 3). In this circuit, the Th2 cytokines IL-4 and IL-13 are also capable of inducing tuft cell hyperplasia (98). In fact, IL-13 produced by activated ILC2s and Th2 cells has been shown to promote an active renewal of IECs, a process that acts as an “epithelial escalator” and helps to expel parasites from the intestine (99). In addition, IL-13 induces GC differentiation, which results in the secretion of mucus and anti-helminthic molecules, such as RELMβ (97).

Zaiss et al. have reported the involvement of AREG, originally described as an epithelial cell-derived factor, which has a critical role in helminth resistance and shows its relevance in the expulsion of the nematodes *T. muris* through the promotion of IEC replacement. Although non-lymphoid gut cells might produce AREG, the exact cellular sources of this molecule are still unknown (100).

Considering a scenario where helminth infection causes the recruitment of different cell populations, it is logical to speculate how ILC2s interplay with phagocytes. In this way, Bouchery et al. (101) reported that ILC2s and T cells cooperate with M2 Mo in the lung during infection with *N. brasiliensis*, thereby trapping and killing the larvae in re-infected mice. Furthermore, they showed that IL-33- or IL-2-dependent ILC2 activation stimulates M2 Mo to reduce worm burden in the lung of re-infected mice. Nevertheless, it is important to emphasize the role of mast cells in the immunity against intestinal helminths, since the accumulation of these cells or mastocytosis is a feature of infections caused by these parasites. The proteases of mast cells promote the breakdown of the narrow joints of the IECs, allowing the discharge of fluids into the intestinal lumen. In line with this, mast cell-deficient mice (by a mutation in the c-kit gene) have been demonstrated to have difficulty in eliminating the *T. spiralis* nematode (102). In addition, a crucial role of mast cells has been recently demonstrated in eradicating *H. polygyrus* in the early stage of infection. These cells secrete IL-33 that activates an ILC2 response after the recognition of tissue damage-derived ATP through the P2X7 receptor (103) (Figure 3).

Finally, a recent mechanism for Th2 immunity constriction has been demonstrated. Early post-infection with *H. polygyrus bakeri*, intestinal lamina propria (LP) cells secrete IL-1β, which inhibits the helminth-induced IL-25 and IL-33 and results in a reduced Th2 protective immune response, thus allowing the parasite chronicity (104) (Figure 3).

## Conclusion

In the last decade, there have been important advances in the knowledge about how helminth parasites are recognized by DCs and also regarding the capacity of these cells to induce a Th2-type response. However, other phagocytes are necessary to generate an intricate network of cytokines, soluble factors, and danger signals arising from the damage produced by parasite migration, which promotes a Th2-type response. This response in turn drives the alternative activation of Mo, and the activation of Eos, basophils, and mast cells, which contribute to the expulsion of intestinal nematodes. Among the phagocytes, M2 Mo play a key role in providing factors for rapid and efficient tissue repair and to complete the process of wound healing.

Although an undeniable involvement of phagocytes, such as Mo, neutrophils, Eos, basophils, mast cells, and antibodies in *in vitro* death mechanisms against helminths, have been demonstrated, it is not clear whether these mechanisms occur *in vivo*. However, there is growing evidence showing the importance of these cells in the mechanisms of protection against parasitic helminths in different settings of cell depletion or vaccination in mice.

Some interactions between phagocytes with other cell types (e.g., ILC2) during helminth infection have been demonstrated. However, further studies are still required to elucidate the factors that modulate the maturation of DCs to promote the Th2 response in naive T cells and possible interaction between other phagocytic cells such as Mo, neutrophils, basophils, and mast cells during infection by helminth parasites.

## Author Contributions

CM and LC wrote the manuscript, LS and DC designed the figures, LSC and MT critically revised the manuscript, and LA and XV revised the manuscript.

## Conflict of Interest Statement

The authors declare that the research was conducted in the absence of any commercial or financial relationships that could be construed as a potential conflict of interest.
